# Real-world efficacy of adjuvant therapy for totally resected stage I lung adenocarcinoma patients with pathological high-risk factors: propensity score analysis

**DOI:** 10.1186/s12893-024-02428-w

**Published:** 2024-05-08

**Authors:** Ke Zhao, Libing Yang, Lei Liu, Guige Wang, Jiaqi Zhang, Xuehan Gao, Chao Guo, Cheng Huang, Yeye Chen, Shanqing Li

**Affiliations:** Department of Thoracic Surgery, Peking Union Medical college Hospital, Chinese Academy of Medical Science & Peking Union Medical College, No.1 Shuaifuyuan, Dongcheng District, Beijing, 100730 China

**Keywords:** Lung adenocarcinoma, Adjuvant therapy, Real-world study, High-risk factors, Propensity score analysis

## Abstract

**Background:**

We investigated the real-world efficacy of adjuvant therapy for stage I lung adenocarcinoma patients with pathological high-risk factors.

**Methods:**

Study participants were enrolled from November 1, 2016 and December 31, 2020. Clinical bias was balanced by propensity score matching. Disease-free survival (DFS) outcomes were compared by Kaplan–Meier analysis. The Cox proportional hazards regression was used to identify survival-associated factors. *p* ≤ 0.05 was the threshold for statistical significance.

**Results:**

A total of 454 patients, among whom 134 (29.5%) underwent adjuvant therapy, were enrolled in this study. One hundred and eighteen of the patients who underwent adjuvant therapy were well matched with non-treatment patients. Prognostic outcomes of the treatment group were significantly better than those of the non-treatment group, as revealed by Kaplan-Meier analysis after PSM. Differences in prevention of recurrence or metastasis between the targeted therapy and chemotherapy groups were insignificant. Adjuvant therapy was found to be positive prognostic factors, tumor size and solid growth patterns were negative.

**Conclusions:**

Adjuvant therapy significantly improved the DFS for stage I lung adenocarcinoma patients with high-risk factors. Larger prospective clinical trials should be performed to verify our findings.

## Introduction

Globally, lung cancer is one of the most common malignant tumors. The International Agency for Research on Cancer (IARC) states that there are about 2.2 million newly diagnosed lung cancer cases every year, and its incidence rate is only second to breast cancer [1]. Lung cancer is also the leading cause of cancer-associated deaths, with about 1.8 million deaths every year [1]. Lung cancer is a group of highly heterogeneous diseases, of which 80–85% belongs to the non-small cell lung cancer (NSCLC) subgroup, and adenocarcinoma is the most common pathological type of NSCLC [2, 3]. 

In 2011, a novel multidisciplinary classification of pulmonary adenocarcinoma was published by the International Association for the Study of Lung Cancer (IASLC), the American Thoracic Society (ATS), and the European Respiratory Society (ERS). Invasive adenocarcinomas are classified with lepidic, acinar, papillary, solid patterns, and micropapillary by predominant patterns [4]. In 2015, spread through air spaces (STAS) was recognized as a new pattern of invasion by the World Health Organization (WHO) [5]. Micropapillary growth patterns, solid growth patterns, STAS, vascular invasion and visceral pleural involvement are negative prognostic factors for lung cancer patients [4, 6–8]. 

In 2015, IASLC updated the TNM classification to inform the treatment of NSCLC patients [9]. The Chinese Society of Clinical Oncology (CSCO) guidelines do not recommend adjuvant treatment for stage I NSCLC patients. Based on findings of the ADAURA study, published guidelines of the National Comprehensive Cancer Network (NCCN) and European Society for Medical Oncology (ESMO) recommend that chemotherapy followed by osimertinib can benefit stage IB patients with high-risk factors [10–14]. 

About 10-27% of stage I NSCLC patients have a probability of dying within five years of diagnosis, which is attributed to tumor recurrence and metastasis [9]. Therefore, there is need to improve on the prognosis of these patients. Some doctors tend to implement adjuvant treatments for patients with high-risk pathological recurrence factors, but the clinical outcomes are unknown. Therefore, we investigated the real-world efficacy of adjuvant therapy for stage I lung adenocarcinoma patients with pathological high-risk factors.

## Methods

### Patient selection and exclusion

This retrospective study was conducted at the Department of Thoracic Surgery in Peking Union Medical College Hospital (PUMCH), Chinese Academy of Medical Sciences and Peking Union Medical College. Electronic medical records for pathological stage I lung cancer patients discharged from PUMCH between November 1, 2016 and December 31, 2020 were continuously retrieved.

Staging was based on IASLC version 8 TNM grading and staging system [9]. Patients should have undergone radical surgery, including lobectomy and elective sublobar resection. In this study, based on the findings of JCOG0804/ WJOG4507L [15], the range of sublobar radical resection for patients was determined. That is, sublobar resection was considered adequate only when the patient’s tumor had a maximum diameter of ≤ 2 cm and the consolidation/tumor ratio (CTR), was ≤ 0.25.

In this study, micropapillary growth patterns, solid growth patterns, STAS, vascular invasion and visceral pleural involvement were treated as pathological high-risk factors for tumor recurrence and metastasis. STAS is defined as tumor cells within airspaces beyond the edge of the main tumor [16]. The 2021 WHO Classification of Thoracic Tumours recommends to document percentages of histologic patterns in invasive lung nonmucinous adenocarcinomas [16], thus, as long as the proportion of micropapillary growth or the solid growth exceeds 5% in the tumor, this high-risk factor is considered to exist by researchers. All patients’ pathologic diagnoses were independently reviewed by 2 pathologists according to the above criteria.

**Fig. 1:**
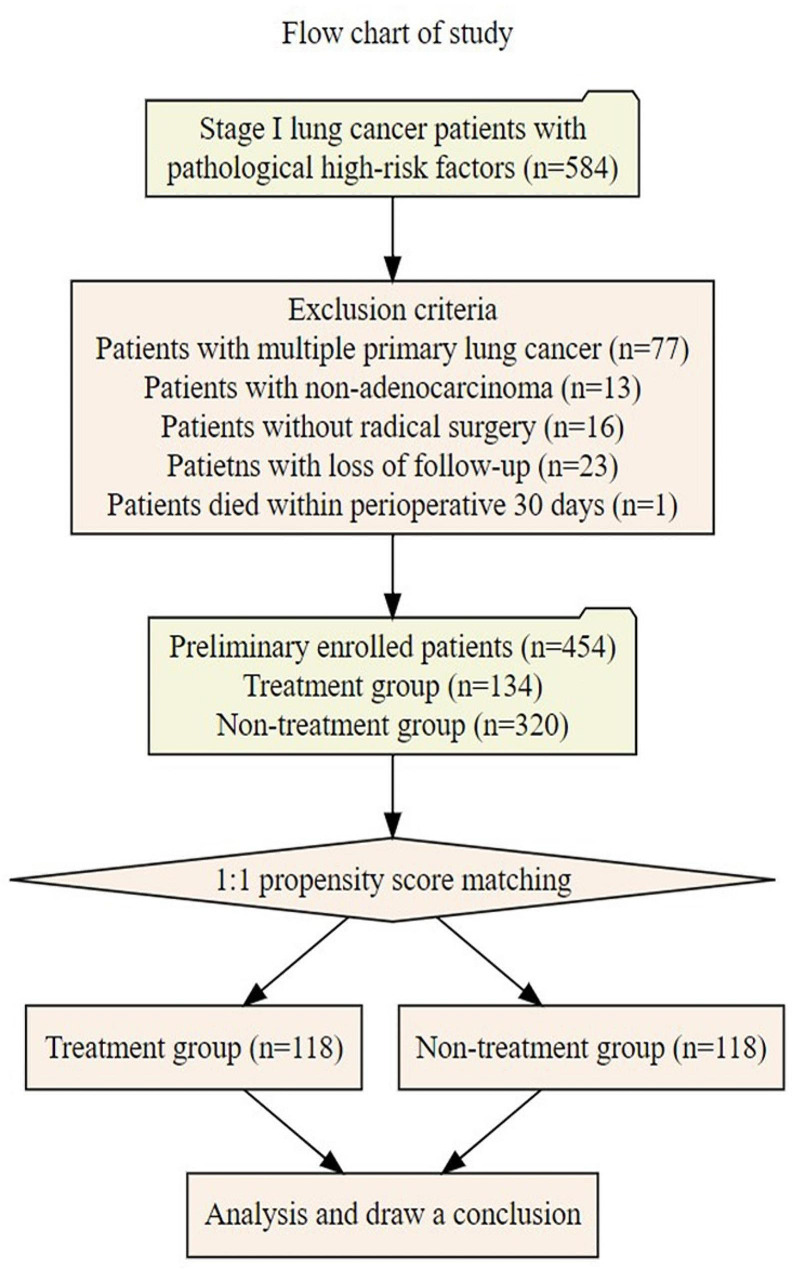
Flow chart of study

Stage I lung cancer patients with any one of the above pathological factors were considered eligible for inclusion, that is, the weights of these factors are consistent. The inclusion criteria and exclusion are shown in Fig. 1. Based on whether they received postoperative adjuvant therapy (including targeted therapy and chemotherapy), patients were assigned into treatment and non-treatment groups.

### Clinical data collection and follow-up

The collected clinical data included sex, age, smoking index (cigarettes per day * years), body mass index, previous comorbidities, tumor size, tumor location, surgical date, surgical method and approach, patients’ pathology reports, adjuvant therapies, disease recurrence or metastasis and death.

Every half a year after surgery, patients were subjected to chest computer tomography (CT) examination. Positron emission tomography (PET)/CT and brain magnetic resonance imaging (MRI) were used to evaluate recurrence and metastasis. Patient follow-up was performed at the outpatient service of the department of thoracic surgery or oncology or by telephone. Disease-free survival (DFS) duration was calculated from the date of surgery until detection of disease progression. Disease progression was indicated by tumor recurrence or metastasis, which were set as the primary endpoints for this study.

### Statistical analysis

Descriptive data for categorical variables are presented as frequencies and proportions and as means, medians, and standard deviations for continuous variables. The Chi-square and Fisher’s exact tests were performed to compare differences between the proportions of categorical variables, while the t-test was used to compare differences between the mean values of continuous variables. Patient backgrounds were matched by propensity score-matching (PSM) analysis using R 4.2.2. Matching was performed in accordance with sex, age, smoke index, ACE-27 scores [17], tumor size, number of risk factors and TNM stage. Nearest neighbor matching with a caliper difference of 0.05 was used to match. The Kaplan–Meier method and log-rank tests were performed to estimate the DFS for the entire cohort. Multivariate analyses were performed using the Cox regression model. Statistical analyses and plotting were performed using SPSS 26.0 and GraphPad Prism 9 softwares. All statistical tests were two-sided, and *p* < 0.05 was the threshold for significance.

## Results

### Patient characteristics before and after PSM

Data for 454 patients were collected. Among them, 134 (29.6%) patients underwent adjuvant therapy while the remaining 320 (70.4%) patients only underwent postoperative regular checks. Baseline characteristics for patients are shown in Table 1. Compared with the non-treatment group before PSM, patients in the treatment group were younger (*p* = 0.013), had bigger tumor sizes (*p* = 0.010), multiple risk factors (*P* = 0.002) and higher pathologic stage (*p* = 0.003) than patients without adjuvant therapy.

**Table 1:** Baseline characteristics of enrolled patients before propensity score matching

Variables	Overall (*n* = 454)	Non-treatment (*n* = 320)	Treatment (*n* = 134)	*p* value
Male, n (%)	173(38.1)	128(40.0)	45(33.6)	0.239
Age group, n (%)				**0.013**
≤ 50 years	64(14.1)	40(12.5)	24(17.9)	
>50 and ≤ 60 years	150(33.0)	99(30.9)	51(38.1)	
>60 and ≤ 70 years	181(39.9)	130(40.6)	51(38.1)	
>70 years	59(13.0)	72(15.6)	8(6.0)	
Smoke index group, n (%)				0.300
No smoking	340(74.9)	236(73.8)	104(77.6)	
Smoking index ≤ 500	49(10.8)	33(10.3)	16(11.9)	
Smoking index > 500	65(14.3)	51(15.9)	14(10.4)	
ACE-27 Score, n (%)				0.955
0	181(39.9)	125(39.1)	56(41.8)	
1	128(28.2)	91(28.4)	37(27.6)	
2	141(31.1)	101(31.6)	40(29.9)	
3	4(0.9)	3(0.9)	1(0.7)	
Diameter group, n (%)				**0.010**
≤1 cm	46(10.1)	37(11.6)	9(6.7)	
>1 and ≤ 2 cm	226(49.8)	169(52.8)	57(42.5)	
>2 and ≤ 3 cm	133(29.3)	87(27.2)	46(34.3)	
>3 and ≤ 4 cm	49(10.8)	27(8.4)	22(16.4)	
Multiple risk factors, n (%)	206(45.4)	130(40.6)	76(56.7)	**0.002**
TNM Stage, n (%)				**0.003**
IA	153(33.7)	122(38.1)	31(23.1)	
IB	301(66.3)	198(61.9)	103(76.9)	

**Fig. 2:**
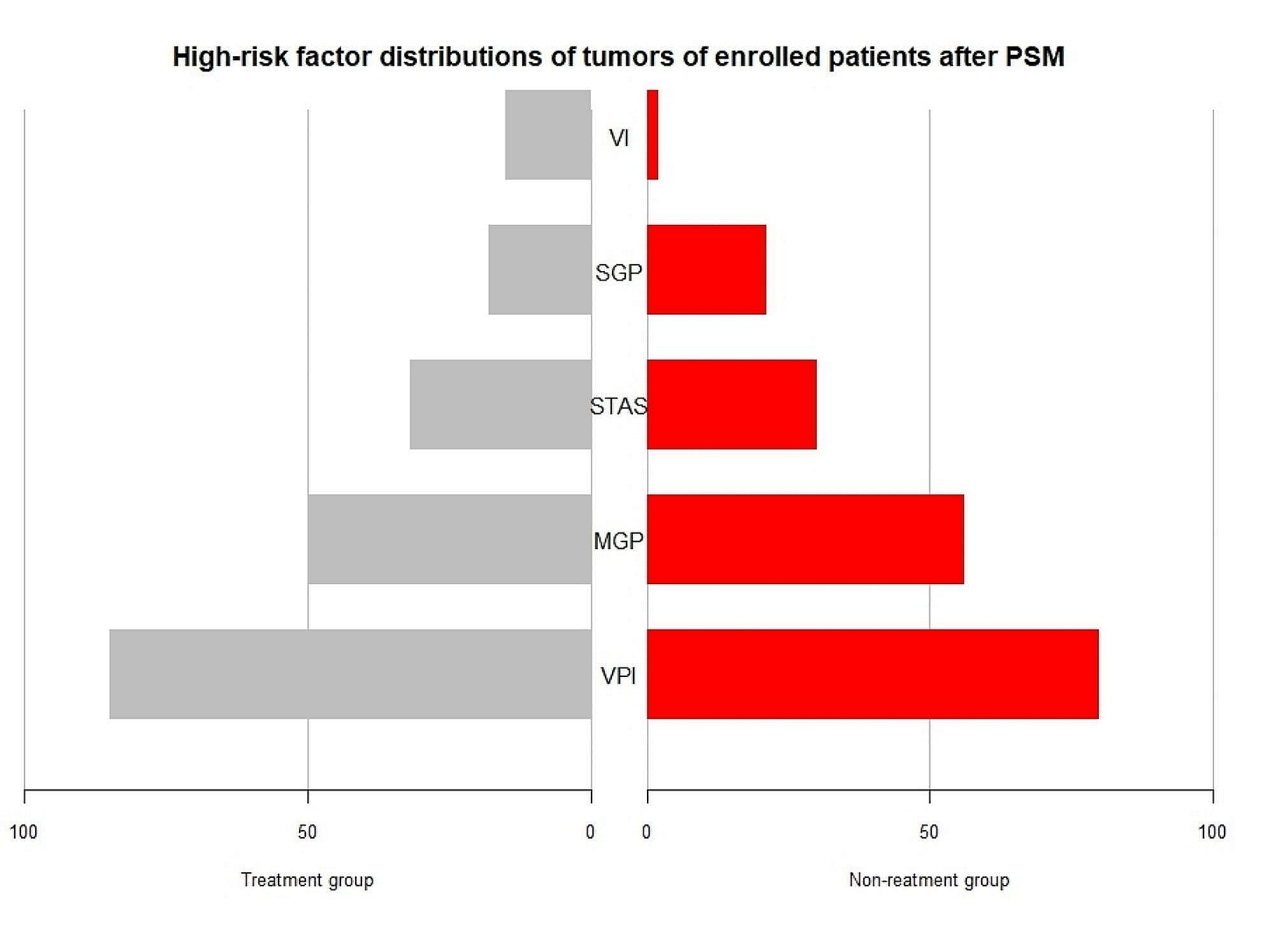
High-risk factor distributions of tumors of enrolled patients after PSM. * VI, vascular invasion; SGP, solid growth patterns; STAS, spread through air spaces; MGP, micropapillary growth patterns; VPI, visceral pleural involvement

Matched characteristics for patients are shown in Table 2. The average age for enrolled patients was 58.5 ± 9.1 years (30–78 years), with male patients accounting for about one third of the total. Distributions of pathological high-risk factors are shown in Fig. 2. There were 118 patients in the treatment group, including 52 patients receiving targeted therapy, 62 patients receiving platinum-based chemotherapy, and four patients receiving chemotherapy followed by targeted treatment. A total of 118 patients in the non-treatment group did not receive any form of adjuvant treatment.

**Table 2:** Baseline characteristics of enrolled patients after propensity score matching

Variables	Overall (*n* = 236)	Non-treatment (*n* = 118)	Treatment (*n* = 118)	*p* value
Male, n (%)	75(31.8)	34(28.8)	41(34.7)	0.402
Age group, n (%)				0.955
≤ 50 years	44(18.6)	23(19.5)	21(17.8)	
>50 and ≤ 60 years	84(35.6)	40(33.9)	44(37.3)	
>60 and ≤ 70 years	92(39.0)	47(39.8)	45(38.1)	
>70 years	16(6.8)	8(6.8)	8(6.8)	
Smoke index group, n (%)				0.749
No smoking	187(79.2)	94(79.7)	93(78.8)	
Smoking index ≤ 500	28(11.9)	15(12.7)	13(11.0)	
Smoking index>500	21(8.9)	9(7.6)	12(10.2)	
ACE-27 Score, n (%)				0.983
0	103(43.6)	53(44.9)	50(42.4)	
1	65(27.5)	32(27.1)	33(28.0)	
2	66(28.0)	32(27.1)	34(28.8)	
3	2(0.8)	1(0.8)	1(0.8)	
Diameter group, n (%)				0.915
≤ 1 cm	17(7.2)	8(6.8)	9(7.6)	
>1 and ≤ 2 cm	104(44.1)	50(42.4)	54(45.8)	
>2 and ≤ 3 cm	80(33.9)	41(34.7)	39(33.1)	
>3 and ≤ 4 cm	35(14.8)	19(16.1)	16(13.6)	
Multiple risk factors, n (%)	121(51.3)	57(48.3)	64(54.2)	0.435
TNM Stage, n (%)				1.000
IA	62(26.3)	31(73.7)	31(73.7)	
IB	174(73.7)	87(73.7)	87(73.7)	

### Clinical and prognostic outcomes before and after PSM

The median follow-up time for all identified patients was 43 months (24–72 months). Prognostic outcomes for the treatment group were better than those of the non-treatment group without significant statistical difference, as revealed by COX analysis before PSM, *p* = 0.098, [hazard ratio (HR) = 0.59, 95% confidence interval (CI): 0.31–1.10]. There were 12 and 47 patients in the two groups who suffered disease recurrence or metastasis, respectively. All stage IA1 patients had no recurrence or metastasis.

**Fig. 3:**
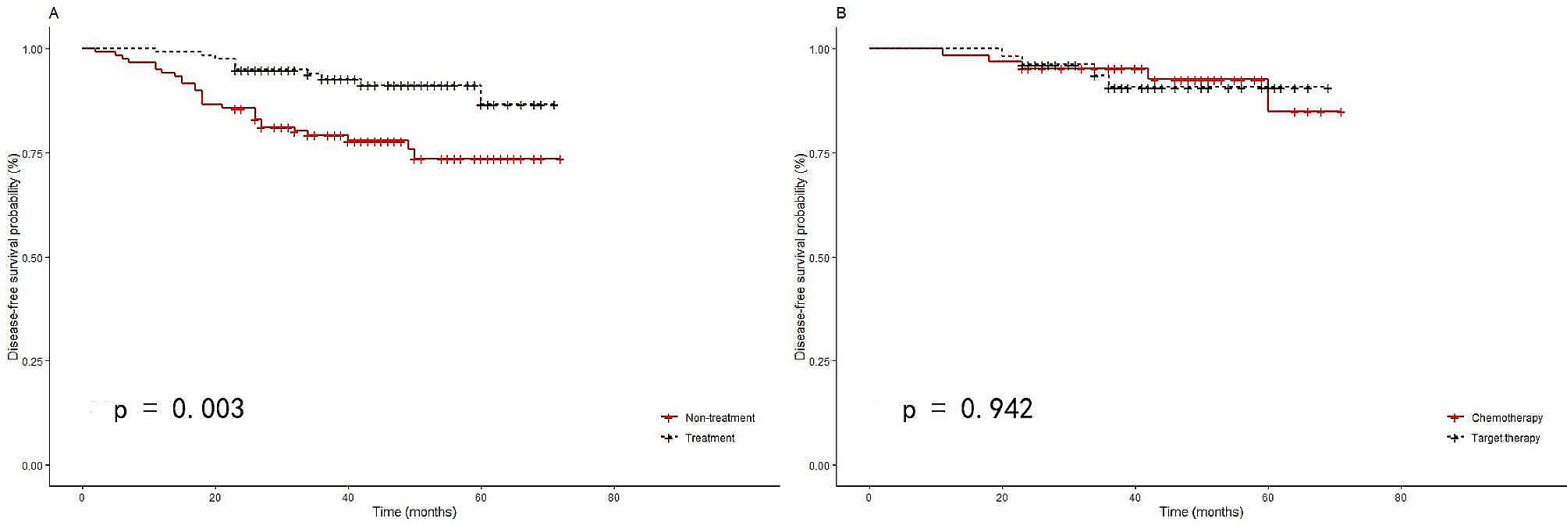
**(A)** The disease-free survival curves for totally resected stage I lung adenocarcinoma patients with pathological high-risk factors with or without adjuvant therapy after propensity score matching, *p* = 0.004. **(B)** The DFS curves for totally resected stage I lung adenocarcinoma patients with pathological high-risk factors with chemotherapy or with targeted therapy after propensity score matching, *p* = 0.942

Disease-free survival outcomes for the treatment group were still significantly better than those of the non-treatment group after PSM, *p* = 0.004, (HR = 0.34, 95% CI: 0.17–0.71) (Fig. 3A). Four and five patients in targeted therapy and chemotherapy groups exhibited disease progression after PSM, respectively. Differences between targeted therapy and chemotherapy in preventing recurrence or metastasis were insignificant, *p* = 0.964, (HR = 0.97, 95% CI: 0.26–3.62) (Fig. 3B).

In subgroup analyses after PSM, we also explored in which subgroups adjuvant therapy would benefit patients more. Patients in the subgroups of women, non-smokers, stage IB, multiple risk factors, age ≤ 50 years, and maximum tumor diameter >2 and ≤ 3 cm showed better benefits from adjuvant treatment (Table 3). Analysis of sex, age, TNM stage, number of risk factors, tumor size, cigarette, adjuvant therapy and different subtype of risk factors by univariate and multivariate COX analysis revealed that adjuvant therapy, tumor size and solid growth patterns are significant prognostic factors (Table 4).

**Table 3:** Subgroups for which adjuvant therapy benefit patients more after propensity score matching

Variables	Univariate analyses HR (95% CI)	*P* value
Female	0.33 (0.13–0.84)	**0.019**
Non-smoker	0.35 (0.16–0.79)	**0.012**
Multiple risk factors	0.27 (0.11–0.70)	**0.007**
Stage IB	0.38 (0.17–0.81)	**0.013**
≤ 50 years	0.20 (0.04–0.92)	**0.039**
>2 and ≤ 3 cm	0.26 (0.08–0.78)	**0.017**

**Table 4:** Univariate and multivariate analysis of DFS after propensity score matching

Variables	Univariate analyses		Multivariate analyses	
	HR (95% CI)	*P* value	HR (95% CI)	*P* value
ACE-27 score	1.16 (0.80–1.69)	0.430		
Age	0.98 (0.95–1.02)	0.321		
Maximum diameter	1.06 (1.02–1.10)	**0.003**	1.05 (1.02–1.09)	**0.005**
Adjuvant therapy	0.34 (0.17–0.71)	**0.004**	0.36 (0.17–0.74)	**0.006**
Visceral pleural involvement	1.14 (0.54–2.43)	0.730		
Micropapillary growth patterns	1.82 (0.95–3.50)	0.071		
Multiple risk factors	1.85 (0.95–3.61)	0.072		
Sex	1.19 (0.61–2.34)	0.611		
Smoking	1.18 (0.54–2.58)	0.684		
Solid growth patterns	3.28 (1.67–6.46)	**0.001**	2.90 (1.46–5.73)	**0.002**
STAS	0.53 (0.20–1.37)	0.190		
TNM stage	1.62 (0.67–3.90)	0.281		
Vascular invasion	1.17 (0.36–3.80)	0.798		

## Discussion

To the best of our knowledge, this is the first report on the real-world efficacy of adjuvant therapy for totally resected stage I lung adenocarcinoma patients with high-risk pathological factors.

The 5-year overall survival (OS) outcomes for stage IA1, IA2, IA3 and IB NSCLC patients have been reported to be 90%, 85%, 80% and 73%, respectively, while the 5-year DFS were 88.2%, 73.6%, 64.5% and 60.1% respectively [9, 18]. These findings imply that there is much room for improvement of the prognosis of stage I NSCLC patients. Unfortunately, previous large-scale studies found patients with stage I NSCLC and good performance status can only benefit from surgery, with efficacies of adjuvant therapies being poor [19–23]. 

In recent years, studies with low levels of evidence have supported the use of adjuvant therapy in selected stage IB NSCLC patients [24–27], which improved the 5-year OS and 5-year DFS of patients. Currently, the ADAURA study is the only large randomized controlled study with good results of targeted therapy for stage IB NSCLC patients [10]. Stage IB NSCLC patients with epidermal growth factor receptor-mutations (EGFRm) who underwent total resection were randomized 1:1 to receive osimertinib or placebo for three years. Compared to the placebo, osimertinib resulted in better DFS outcomes, regardless of whether patients received adjuvant chemotherapy (DFS HR = 0.16, 95% CI: 0.10–0.26) or not (HR = 0.23, 95% CI: 0.13–0.40). The NCCN and ESMO guidelines note the above outcomes [11, 13]. Based on these findings, some of the patients with high-risk pathological factors have also been subjected to individualized adjuvant therapies, with positive outcomes.

In this study, 454 patients were enrolled, among whom 134 (29.5%) patients received adjuvant treatment while the other 320 (70.5%) patients did not. We found that adjuvant treatment didn’t improved the DFS of patients, *p* = 0.098, however, we believe that this result does not really reflect the role of adjuvant therapy. This is because baseline characteristics for the two groups did not match, and since adjuvant therapy for stage I patients is not the standard treatment, clinicians only tend to recommend it for patients with a high probability of disease progression. In this study, tumor sizes for patients in the treatment group were markedly larger, significantly more patients had multiple risk factors, percentage of younger patients were higher, and pathological staging was higher before PSM (Table 1). Therefore, we tried to eliminate these differences by PSM. After PSM, baseline characteristics of the two groups were balanced (Table 2) and adjuvant treatment significantly reduced the risks of recurrence and metastasis, *p* = 0.004.

Since the protocols for adjuvant treatments, including chemotherapy alone, targeted therapy alone, and chemotherapy followed by targeted therapy differ, we analyzed whether different protocols result in differences in improving the prognostic outcomes of patients. The number of patients undergoing chemotherapy followed by targeted therapy is small, thus, we analyzed the advantages and disadvantages of the first two protocols. The patients in the chemotherapy alone group received platinum-doublet chemotherapy for at least 4 cycles, who in the targeted therapy alone group received first generation tyrosine kinase inhibitors (TKI) for at least 24 months. Both protocols improved the DFS of patients, however, differences in therapeutic effects between the two protocols were insignificant. Selection of specific treatment plans for patients should be based on characteristics of the two protocols.

Targeted therapy, represented by TKI, has the advantages of strong pertinence, less side effects and convenient drug delivery. It should also be noted that because of the failure of matching drive genes, nearly 40% of NSCLC patients cannot receive targeted therapy [28, 29], who need to adopt other treatment methods to reduce the likelihood of recurrence and metastasis. Moreover, because of the high-cost implications and long treatment cycle, the economic burden to patients may be huge. Third generation TKIs are not cost-effective as first-line therapeutic options for EGFRm NSCLC [30], and osimertinib first-line treatment is less cost-effective than first generation TKIs such as gefitinib or erlotinib first-line and gene-guided osimertinib second-line strategies in China [31]. As a traditional cancer treatment method, chemotherapy has the advantages of quick effect, short treatment period and low price [32]. However, it can kill cancer and normal cells in tissues, resulting in more side effects, thus, patients undergoing chemotherapy should also be closely monitored in hospital. We suggest that administration of adjuvant therapies should take into account multiple factors such as medical benefits, cost-effectiveness, and accessibility among others, and be decided by doctors and patients through consultations.

In current study, some patients chose to postpone adjuvant treatment until the recurrence did occur, which can lead to some patients entering the advanced stage of lung cancer early based on the results of this research. For patients in the treatment group, they started adjuvant therapy beforehand. When the disease progresses, they will have to receive second-line systemic treatment, such as third-generation TKI or immunotherapy, which would result in higher costs. The cost-benefit analysis of adjuvant therapy for patients with stage I lung adenocarcinoma has not been searched at present, so when is the best time to start adjuvant therapy and how to minimize patient expenses as much as possible need to be further explored.

In subgroup analysis after PSM, we found that patients in the subgroups of women, non-smokers, stage IB, multiple risk factors, age ≤ 50 years, and maximum tumor diameter >2 and ≤ 3 cm groups were more likely to benefit from adjuvant therapies. COX regression analysis revealed that the adjuvant therapy, tumor size and solid growth patterns were significant prognostic factors for all enrolled patients. Patients with larger tumors and solid growth patterns are more likely to develop recurrence or metastasis, and adjuvant therapy can prevent disease progression.

Based on these findings, we recommend chemotherapy or targeted therapy in totally resected stage I lung adenocarcinoma patients with pathological high-risk factors, especially those who meet the following criteria: (i) Presence of two or more of the five high-risk factors in tumors, including micropapillary growth patterns, solid growth patterns, STAS, vascular invasion as well as visceral pleural involvement; (ii) Patients with stage IB cancer; (iii) Non-smoking patients, (iv) Female patients; (v) Patients with age ≤ 50 years; and (vi) Patients with maximum tumor diameter >2 and ≤ 3 cm.

This study has some limitations. First, its retrospective nature leads to an inevitable selection bias. Second, some clinicopathological information, including gene mutation status, and manifestations of tumor in chest CT among others were not collected, which may have affected our comprehensive assessment of patient diagnosis and treatment outcomes. In addition, disease progression incidences in stage I lung adenocarcinoma patients is relatively lower, therefore, it is difficult to obtain accurate data for median DFS, leading us to qualitatively describe the improvement of patients’ prognosis. Finally, the follow-up time for the enrolled patients was not sufficient, and the median follow-up time was only 30 months. Although the short-term follow-up time was corrected by mathematical modeling of the statistical analysis package, due to some deviations, the conclusions must be carefully explained. Prospective clinical randomized controlled trials should be performed to confirm our findings.

## Data Availability

The datasets used and/or analyzed during the current study available from the corresponding author on reasonable request.

## References

[1] Sung H, Ferlay J, Siegel RL, Laversanne M, Soerjomataram I, Jemal A, Bray F (2021). Global Cancer statistics 2020: GLOBOCAN estimates of incidence and Mortality Worldwide for 36 cancers in 185 countries. Cancer J Clin.

[2] Warth A, Muley T, Kossakowski C, Stenzinger A, Schirmacher P, Dienemann H, Weichert W (2015). Prognostic impact and clinicopathological correlations of the cribriform pattern in pulmonary adenocarcinoma. J Thorac Oncology: Official Publication Int Association Study Lung Cancer.

[3] Warth A, Muley T, Meister M, Stenzinger A, Thomas M, Schirmacher P, Schnabel PA, Budczies J, Hoffmann H, Weichert W (2012). The novel histologic International Association for the study of Lung Cancer/American Thoracic Society/European Respiratory Society classification system of lung adenocarcinoma is a stage-independent predictor of survival. J Clin Oncology: Official J Am Soc Clin Oncol.

[4] Travis WD, Brambilla E, Noguchi M, Nicholson AG, Geisinger KR, Yatabe Y, Beer DG, Powell CA, Riely GJ, Van Schil PE (2011). International association for the study of lung cancer/american thoracic society/european respiratory society international multidisciplinary classification of lung adenocarcinoma. J Thorac Oncology: Official Publication Int Association Study Lung Cancer.

[5] Travis WD, Brambilla E, Nicholson AG, Yatabe Y, Austin JHM, Beasley MB, Chirieac LR, Dacic S, Duhig E, Flieder DB (2015). The 2015 World Health Organization Classification of Lung Tumors: impact of genetic, clinical and radiologic advances since the 2004 classification. J Thorac Oncology: Official Publication Int Association Study Lung Cancer.

[6] Shimada Y, Saji H, Yoshida K, Kakihana M, Honda H, Nomura M, Usuda J, Kajiwara N, Ohira T, Ikeda N (2012). Pathological vascular invasion and tumor differentiation predict cancer recurrence in stage IA non-small-cell lung cancer after complete surgical resection. J Thorac Oncology: Official Publication Int Association Study Lung Cancer.

[7] Lakha S, Gomez JE, Flores RM, Wisnivesky JP (2014). Prognostic significance of visceral pleural involvement in early-stage lung cancer. Chest.

[8] Kadota K, Nitadori JI, Sima CS, Ujiie H, Rizk NP, Jones DR, Adusumilli PS, Travis WD (2015). Tumor Spread through Air Spaces is an important pattern of Invasion and impacts the frequency and location of recurrences after Limited Resection for Small Stage I Lung Adenocarcinomas. J Thorac Oncology: Official Publication Int Association Study Lung Cancer.

[9] Goldstraw P, Chansky K, Crowley J, Rami-Porta R, Asamura H, Eberhardt WE, Nicholson AG, Groome P, Mitchell A, Bolejack V (2016). The IASLC Lung Cancer Staging Project: proposals for revision of the TNM Stage groupings in the Forthcoming (Eighth) Edition of the TNM classification for Lung Cancer. J Thorac Oncology: Official Publication Int Association Study Lung Cancer.

[10] Wu YL, John T, Grohe C, Majem M, Goldman JW, Kim SW, Kato T, Laktionov K, Vu HV, Wang Z (2022). Postoperative chemotherapy use and outcomes from ADAURA: Osimertinib as Adjuvant Therapy for Resected EGFR-Mutated NSCLC. J Thorac Oncology: Official Publication Int Association Study Lung Cancer.

[11] Network NCC. NCCN Clinical Practice Guidelines in Oncology of Non-Small Cell Lung Cancer Version 1.2023.; 2022.

[12] Oncology CSC. Guidelines for diagnosis and treatment of non-small cell lung cancer.; 2022.

[13] Remon J, Soria JC, Peters S (2021). Early and locally advanced non-small-cell lung cancer: an update of the ESMO Clinical Practice Guidelines focusing on diagnosis, staging, systemic and local therapy. Annals Oncology: Official J Eur Soc Med Oncol.

[14] Postmus PE, Kerr KM, Oudkerk M, Senan S, Waller DA, Vansteenkiste J, Escriu C, Peters S (2017). Early and locally advanced non-small-cell lung cancer (NSCLC): ESMO Clinical Practice guidelines for diagnosis, treatment and follow-up. Annals Oncology: Official J Eur Soc Med Oncol.

[15] Suzuki K, Watanabe S, Wakabayashi M, Moriya Y, Yoshino I, Tsuboi M, Mitsudomi T, Asamura H. A nonrandomized confirmatory phase III study of sublobar surgical resection for peripheral ground glass opacity dominant lung cancer defined with thoracic thin-section computed tomography (JCOG0804/WJOG4507L). J Clin Oncol 2017, 35(15).

[16] Nicholson AG, Tsao MS, Beasley MB, Borczuk AC, Brambilla E, Cooper WA, Dacic S, Jain D, Kerr KM, Lantuejoul S (2022). The 2021 WHO classification of lung tumors: impact of advances since 2015. J Thorac Oncology: Official Publication Int Association Study Lung Cancer.

[17] Piccirillo JF, Tierney RM, Costas I, Grove L, Spitznagel EL (2004). Prognostic importance of comorbidity in a hospital-based cancer registry. JAMA.

[18] Okami J, Shintani Y, Okumura M, Ito H, Ohtsuka T, Toyooka S, Mori T, Watanabe SI, Date H, Yokoi K (2019). Demographics, Safety and Quality, and Prognostic Information in both the Seventh and Eighth editions of the TNM classification in 18,973 Surgical cases of the Japanese Joint Committee of Lung Cancer Registry Database in 2010. J Thorac Oncology: Official Publication Int Association Study Lung Cancer.

[19] Scagliotti GV, Fossati R, Torri V, Crinò L, Giaccone G, Silvano G, Martelli M, Clerici M, Cognetti F, Tonato M (2003). Randomized study of adjuvant chemotherapy for completely resected stage I, II, or IIIA non-small-cell lung cancer. J Natl Cancer Inst.

[20] Douillard JY, Rosell R, De Lena M, Carpagnano F, Ramlau R, Gonzáles-Larriba JL, Grodzki T, Pereira JR, Le Groumellec A, Lorusso V (2006). Adjuvant vinorelbine plus cisplatin versus observation in patients with completely resected stage IB-IIIA non-small-cell lung cancer (Adjuvant Navelbine International Trialist Association [ANITA]): a randomised controlled trial. Lancet Oncol.

[21] Pignon JP, Tribodet H, Scagliotti GV, Douillard JY, Shepherd FA, Stephens RJ, Dunant A, Torri V, Rosell R, Seymour L (2008). Lung adjuvant cisplatin evaluation: a pooled analysis by the LACE Collaborative Group. J Clin Oncology: Official J Am Soc Clin Oncol.

[22] Zhong WZ, Wang Q, Mao WM, Xu ST, Wu L, Shen Y, Liu YY, Chen C, Cheng Y, Xu L (2018). Gefitinib versus vinorelbine plus cisplatin as adjuvant treatment for stage II-IIIA (N1-N2) EGFR-mutant NSCLC (ADJUVANT/CTONG1104): a randomised, open-label, phase 3 study. Lancet Oncol.

[23] He J, Su C, Liang W, Xu S, Wu L, Fu X, Zhang X, Ge D, Chen Q, Mao W (2021). Icotinib versus chemotherapy as adjuvant treatment for stage II-IIIA EGFR-mutant non-small-cell lung cancer (EVIDENCE): a randomised, open-label, phase 3 trial. Lancet Respiratory Med.

[24] Zhou K, Zhao Y, Liang L, Cao J, Lin H, Peng Z, Mei J (2022). Adjuvant chemotherapy may improve long-term outcomes in stage IB non-small cell lung cancer patients with previous malignancies: a propensity score-matched analysis. Front Oncol.

[25] Xu Y, Wan B, Zhu S, Zhang T, Xie J, Liu H, Zhan P, Lv T, Song Y (2021). Effect of adjuvant chemotherapy on survival of patients with 8th Edition Stage IB Non-small Cell Lung Cancer. Front Oncol.

[26] Zhang P, Duan J, Bai H, Wang Z, Gao S, Tan F, Gao Y, Wang X, Wan R, Xu J (2021). Influence of adjuvant chemotherapy on survival for patients with stage IB and IIA non-small cell lung cancer. Thorac cancer.

[27] Lin C, Hu F, Chu H, Ren P, Ma S, Wang J, Bai J, Han X, Ma S (2021). The role of EGFR-TKIs as adjuvant therapy in EGFR mutation-positive early-stage NSCLC: a meta-analysis. Thorac cancer.

[28] da Cunha Santos G, Shepherd FA, Tsao MS (2011). EGFR mutations and lung cancer. Annu Rev Pathol.

[29] Tan CS, Kumarakulasinghe NB, Huang YQ, Ang YLE, Choo JR, Goh BC, Soo RA (2018). Third generation EGFR TKIs: current data and future directions. Mol Cancer.

[30] Aguiar PN, Haaland B, Park W, San Tan P, Del Giglio A, de Lima Lopes G (2018). Cost-effectiveness of Osimertinib in the First-Line treatment of patients with EGFR-Mutated Advanced Non-small Cell Lung Cancer. JAMA Oncol.

[31] Cai H, Zhang L, Li N, Chen S, Zheng B, Yang J, Weng L, Liu MB (2019). Cost-effectiveness of Osimertinib as First-line treatment and sequential therapy for EGFR mutation-positive non-small cell Lung Cancer in China. Clin Ther.

[32] Shi Y, Pei R, Liu S (2022). Osimertinib versus platinum-pemetrexed in patients with previously treated EGFR T790M advanced non-small cell lung cancer: an updated AURA3 trial-based cost-effectiveness analysis. Front Oncol.

